# Hypercoagulability Predicts Survival and Reflects NET-Associated Thromboinflammation in Advanced Pancreatic Cancer

**DOI:** 10.3390/cancers18132120

**Published:** 2026-06-30

**Authors:** Lingaku Lee, Masami Miki, Masayuki Hijioka, Terumasa Hisano, Rie Sugimoto, Masayuki Furukawa

**Affiliations:** Department of Hepato-Biliary-Pancreatology, NHO Kyushu Cancer Center, Fukuoka 811-1395, Japan

**Keywords:** advanced pancreatic cancer, cancer-associated thrombosis, hypercoagulability, neutrophil extracellular traps, venous thromboembolism

## Abstract

**Background**: Cancer-associated thrombosis is a major complication in pancreatic cancer; however, its true burden and prognostic significance remain unclear, largely owing to under-detection of asymptomatic events. In addition, the clinical relevance of cancer-related hypercoagulability and neutrophil extracellular traps (NETs), which may link thrombosis and tumor biology, has not been adequately evaluated in advanced pancreatic cancer. **Methods**: In this prospective study, newly diagnosed patients with unresectable pancreatic ductal adenocarcinoma underwent systematic screening for venous thromboembolism (VTE) at baseline, followed by longitudinal surveillance. Circulating coagulation markers and NET-related biomarkers were analyzed. Associations among VTE, hypercoagulability, NET-related biomarkers, and overall survival (OS) were evaluated. **Results**: Among 134 patients, VTE was detected at diagnosis in 28.4%, with the majority being asymptomatic. Hypercoagulability and NET-related biomarkers were significantly associated with VTE occurrence. Patients with baseline VTE (6.2 vs. 12.1 months, *p* = 0.002) and hypercoagulability (7.7 vs. 15.2 months, *p* = 0.002) demonstrated shorter OS. In multivariate analysis, hypercoagulability, but not baseline VTE, remained independently associated with inferior OS (hazard ratio 2.03, 95% confidence interval 1.27–3.27). Notably, the adverse prognostic impact of hypercoagulability was consistently observed across nearly all predefined clinical subgroups. Furthermore, a reduction in or normalization of D-dimer levels following anticoagulant therapy was associated with prolonged survival. **Conclusions**: Hypercoagulability, rather than overt thrombotic events, independently predicts survival outcomes in advanced pancreatic cancer. These findings support a biology-driven approach to thrombosis assessment and indicate that monitoring and therapeutic modulation of hypercoagulability may improve risk stratification and clinical management in this population.

## Simple Summary

Patients with advanced pancreatic cancer are at a particularly high risk of developing venous thromboembolism, which can interrupt cancer treatment and worsen clinical outcomes. However, the true frequency and clinical significance of these clotting complications remain unclear because previous studies mainly relied on clinically apparent events and may have overlooked asymptomatic cases. In this prospective study, all patients underwent systematic screening for venous thromboembolism together with comprehensive evaluation of blood coagulation and inflammation-related biomarkers. We found that venous thromboembolisms were common and were frequently asymptomatic. More importantly, persistent activation of the blood coagulation system, rather than the presence of blood clots alone, was strongly associated with worse survival. Markers related to neutrophil extracellular traps were also associated with clot formation and coagulation activation. These findings provide new insight into the biological link between thrombosis and tumor progression and may support more personalized risk assessment and treatment strategies in pancreatic cancer.

## 1. Introduction

Cancer-associated thrombosis (CAT) is a major and potentially life-threatening complication in patients with malignancy and is the second leading cause of death in this population [1]. In addition to its direct morbidity, CAT frequently disrupts anticancer therapy, increases unplanned hospitalizations, and substantially worsens quality of life [1,2,3]. The risk of CAT increases with advancing disease stage [2,4], with pancreatic cancer being one of the highest-risk solid tumors [2,4,5,6]. Advanced pancreatic cancer (APC) in particular has an extremely poor prognosis despite recent therapeutic advances [7,8]. In this context, thrombotic complications may further compromise survival outcomes and limit treatment options. Therefore, early identification of patients at high risk of thrombosis and timely implementation of appropriate therapeutic interventions are of critical importance in the management of APC.

Several studies have reported the occurrence of CAT in APC; however, reported incidence rates vary widely across studies, ranging from 5% to 42% [7,8,9,10,11,12,13,14,15]. This variation appears largely attributable to differences in study design, particularly whether thrombosis was the primary focus of investigation or assessed within the context of chemotherapeutic trials [16]. In routine clinical practice, asymptomatic CAT is frequently overlooked because thrombotic events are typically identified only when clinically suspected or incidentally detected [16,17]. Consequently, retrospective studies relying on clinically apparent events [9,10,11,12,13,14,15], as well as the reporting of adverse events in chemotherapeutic clinical trials [7,8], are likely to underestimate the true incidence of CAT [11,16]. Accurate assessment of the thrombotic burden requires a prospective design incorporating systematic venous thromboembolism (VTE) screening in all participants. However, the limited number of adequately powered prospective investigations in APC restricts a comprehensive understanding of the true thrombotic burden and its clinical impact in advanced disease.

The prognostic impact of CAT in patients with APC remains controversial. Several studies have demonstrated that baseline or early-onset CAT is associated with poorer survival [11,15,18,19], whereas others have reported no independent association between CAT and overall survival (OS) [10,13,14]. The likely underestimation of CAT incidence in these studies, as mentioned earlier, leaves its true prognostic significance uncertain. Hypercoagulability, characterized by elevated D-dimer levels and abnormalities in other coagulation parameters, has likewise been suggested to correlate with adverse outcomes [6,20,21]; however, the strength and independence of this association remain incompletely defined in patients with APC, particularly in real-world clinical settings. Neutrophil extracellular traps (NETs) have emerged as a key contributor to CAT [22,23,24]. In addition to promoting thrombus formation, accumulating evidence indicates that NETs may facilitate tumor progression, metastasis, and cancer-related inflammation, highlighting their potential as a therapeutic target in multiple types of malignancies [22,25] including pancreatic cancer [26,27]. Nevertheless, clinical evidence evaluating the relevance of NETs in patients with APC remains limited.

We therefore conducted a prospective study to define the true burden of CAT and hypercoagulability in APC, to evaluate the role of NETs in CAT development, and to determine the prognostic impact of these thrombotic and coagulation abnormalities. We hypothesized that underlying hypercoagulability and NET activation are associated with increased thrombotic burden and poorer survival in APC patients.

## 2. Materials and Methods

### 2.1. Patients and Study Design

Patients with newly diagnosed, histologically or cytologically confirmed unresectable pancreatic ductal adenocarcinoma were prospectively enrolled between July 2020 and June 2022. Unresectable disease was defined as either metastatic disease or locally advanced disease with major vascular invasion precluding curative surgical resection. Exclusion criteria included age ≤ 18 years, tumor recurrence after prior curative resection, and concomitant or previous malignancy within the preceding 5 years. All enrolled patients underwent baseline screening for VTE, including Doppler ultrasonography for deep vein thrombosis and contrast-enhanced computed tomography (CT) for pulmonary embolism. During the observation period, contrast-enhanced CT was performed every 2–3 months as part of routine clinical practice, and D-dimer levels were measured monthly or whenever symptoms suggestive of VTE developed. Patients were prospectively followed until the data cutoff date (December 2023).

The primary objective of this study was to evaluate the prognostic impact of hypercoagulability in APC. Secondary objectives included identification of factors associated with hypercoagulability and VTE occurrence, with particular focus on NET activation, as well as performance assessment of established VTE risk assessment models (RAMs) in an APC population.

### 2.2. Sample Collection and ELISA

Baseline blood samples were obtained from all patients, and additional samples were collected at the time of newly diagnosed VTE during follow-up. Blood samples were centrifuged at 1500× *g* for 15 min within 1 h of collection to obtain platelet-poor plasma and stored in aliquots at −80 °C until analysis.

Cell-free DNA was extracted from plasma using a MagMAX^TM^ Cell-Free DNA Isolation Kit (Applied Biosystems, Waltham, MA, USA) and quantified using a Quant-iT^TM^ PicoGreen^TM^ dsDNA kit (ThermoFisher Scientific, Waltham, MA, USA). Enzyme-linked immunosorbent assays (ELISAs) were used according to manufacturers’ instructions to determine plasma levels of soluble P-selectin, tissue factor, carbohydrate antigen 125 (CA125), myeloperoxidase (all R&D Systems, Minneapolis, MN, USA), prothrombin fragment 1+2 (PF1+2; Siemens Healthcare Diagnostics, Munich, Germany), and calprotectin (Hycult Biotech, Uden, The Netherlands). Other laboratory findings were obtained from data collected during routine clinical practice.

### 2.3. Data Collection and Definition

Data collected from electronically stored medical records included patient characteristics (age, sex, body mass index, and Eastern Cooperative Oncology Group [ECOG] Performance Status [PS]), radiological findings (location of primary tumor, stage, and major vascular invasion), and clinical (medical history and comorbidities) and laboratory (blood count, C-reactive protein [CRP], thrombin–antithrombin III complex [TAT], plasmin–α2 plasmin inhibitor complex [PIC], carcinoembryonic antigen [CEA], and carbohydrate antigen 19–9 [CA19-9]) findings.

Scores for VTE RAMs, including Khorana [5], Vienna [28], PROTECHT [29], CONKO [30], ONKOTEV [31], COMPASS [32], and RIETE [33], were calculated as previously reported. The components and scoring algorithms of these RAMs are detailed in Supplementary Table S1.

Inflammation was defined as an elevation of either the baseline white blood cell count or CRP above predefined cutoff values. NET-related biomarker positivity was defined as an elevation of at least one NET-related biomarker (cell-free DNA, calprotectin, or myeloperoxidase). Hypercoagulability was defined as an elevation of at least one coagulation-related biomarker (tissue factor, PF1+2, TAT, D-dimer, or PIC) above predefined cutoff values.

Among patients with baseline VTE treated with anticoagulants, longitudinal changes in D-dimer levels were evaluated in relation to overall survival. For these analyses, a “continuous decrease” was defined as a progressive decline in D-dimer levels at every consecutive assessment during the first 3–6 months of anticoagulant therapy, whereas “normalization” was defined as achievement of a D-dimer level below 0.5 μg/mL during follow-up.

### 2.4. Ethical Standards

This study was conducted in accordance with the ethical principles outlined in the 1964 Declaration of Helsinki and its subsequent amendments. All the participants gave written informed consent. The study protocol was approved by the Institutional Review Board of the NHO Kyushu Cancer Center (approval number; 2020-18) and registered in the University Hospital Medical Information Network clinical trials registry of Japan (registration number; UMIN000040965).

### 2.5. Statistics

OS was defined as the interval from diagnosis to death from any cause or to the last follow-up. The OS and cumulative incidence of VTE were estimated using the Kaplan–Meier method and compared using the log-rank test. The Cox proportional hazard model was used to calculate hazard ratios (HRs) and 95% confidence intervals (CIs). Differences between two groups were compared using Student’s t-test for ordinal variables and Fisher’s exact test for categorical variables. Receiver operating characteristic curve analysis was performed to determine the optimal cutoff values for biomarkers predicting VTE occurrence based on maximal sensitivity and specificity. The cutoff value of the D-dimer used for survival analyses (4.45 μg/mL) was determined by ROC analysis, whereas the cutoff value used for VTE analyses (1.44 μg/mL) was based on the threshold incorporated in the Khorana score [5]. Prognostic factors for OS were evaluated using a multivariable Cox regression model. Variables with *p*-values < 0.05 in univariable analyses were considered candidates for inclusion in the multivariable model. All statistical tests were two-sided, and *p*-values < 0.05 were considered statistically significant. Statistical analyses were performed using Prism version 10.6 (GraphPad Software, San Diego, CA, USA).

## 3. Results

### 3.1. Patient Characteristics and Prevalence of VTE

A total of 134 consecutive patients were prospectively enrolled. Baseline demographic and clinical characteristics are summarized in Table 1.

**Figure 1:**
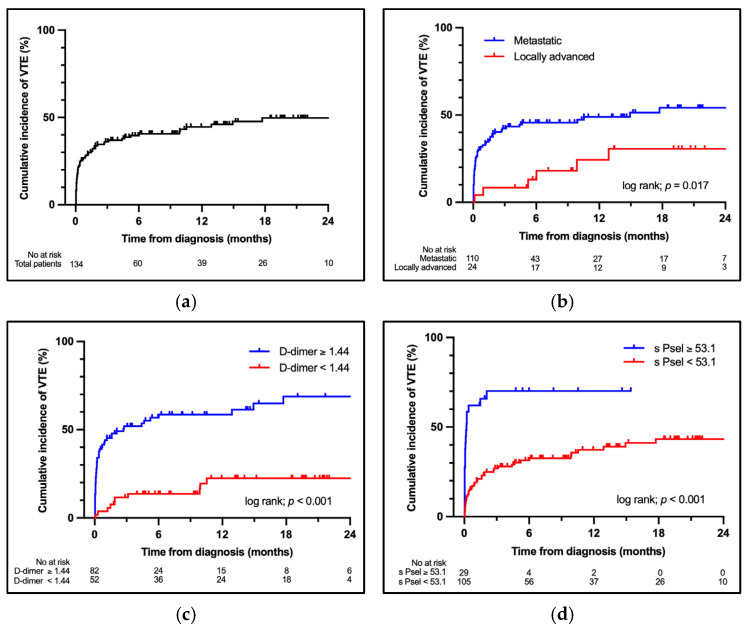
Kaplan–Meier curves for VTE occurrence. (**a**) Probability of VTE occurrence in all enrolled patients. (**b**–**d**) Probability of VTE occurrence according to disease extent (**b**), baseline D-dimer levels (**c**), and soluble P-selectin levels (**d**). s Psel, soluble P-selectin; VTE, venous thromboembolism.

The cohort comprised 50.0% males, with a median age of 69 years (range, 40–88). Most patients had an ECOG PS of 0–1 (86.6%) and metastatic disease (82.1%). VTE was detected in 38 patients (28.4%) at baseline and as newly developed in 22 patients (15.9%) during the observation period (Supplementary Table S2). Most VTE events were asymptomatic (81.7%) and involved distal deep vein thrombosis (83.3%). No VTE-related deaths occurred; however, anticancer treatment delay or discontinuation because of VTE was observed in four patients (10.5%) at baseline and in one patient (4.5%) during follow-up. Among the 38 patients with baseline VTE, 33 received direct oral anticoagulants (DOACs). Six patients received induction therapy with unfractionated heparin before transitioning to DOACs. In addition, two patients received antiplatelet agents and one patient received a vitamin K antagonist (Supplementary Table S2). The cumulative incidence of VTE at 3 and 6 months was 36.2% and 39.7%, respectively, reaching a plateau thereafter (Figure 1a).

Baseline VTE prevalence was significantly higher in patients with poor PS (23.3% vs. 61.1%; *p* = 0.003) and metastatic disease (8.3% vs. 32.7%; *p* = 0.022) (Table 1 and Figure 1b). Among variables incorporated in VTE risk assessment models (RAMs), elevated D-dimer (3.6% vs. 46.2%; *p* < 0.001) and soluble P-selectin levels (19.1% vs. 62.1%; *p* < 0.001) were strongly associated with baseline VTE. Similar associations were observed for cumulative VTE incidence (Figure 1c,d). Other clinical and laboratory variables were not significantly associated with VTE occurrence (Table 1).

### 3.2. VTE Risk Assessment Models

**Figure 2:**
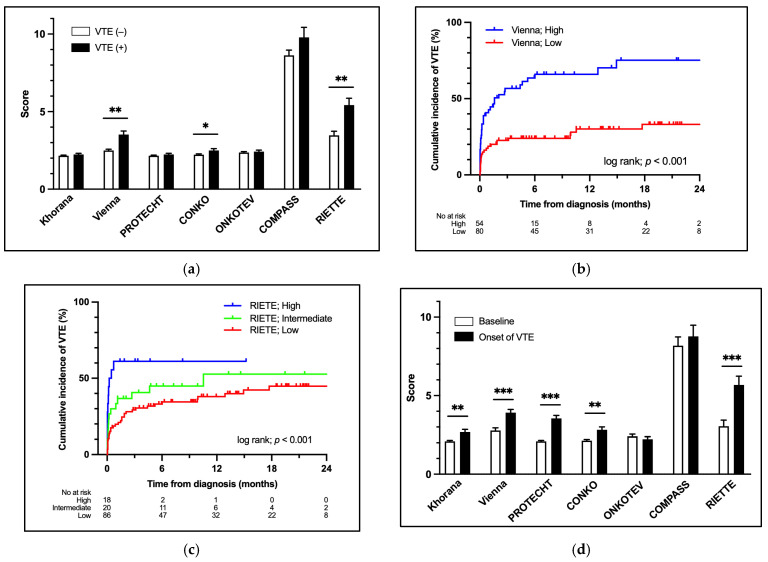
Utility of VTE risk assessment models in APC. Probability of VTE occurrence according to baseline Vienna (**a**) and RIETE (**b**) scores. (**c**) Difference in risk assessment model scores between patients with and without VTE at baseline. (**d**) Change in risk assessment model scores from baseline to the onset of VTE during follow-up. *: *p* < 0.05, **: *p* < 0.01, and ***: *p* < 0.001. VTE, venous thromboembolism.

Higher scores in the Vienna (18.8% vs. 42.6%; *p* = 0.003), CONKO (23.0% vs. 44.1%; *p* = 0.027), and RIETE (19.8% vs. 33.3% vs. 61.1%; *p* < 0.001) models were significantly associated with increased baseline VTE prevalence, whereas other RAMs demonstrated limited predictive performance (Table 2, Figure 2a). Comparable findings were observed for cumulative VTE incidence (Table 2, Figure 2b,c). Among patients who developed VTE during follow-up, RAM scores assessed at VTE onset were significantly increased compared with baseline values, except for the ONKOTEV and COMPASS models (Figure 2d).

### 3.3. Biochemical and Coagulation Parameters Associated with VTE

Patients with VTE exhibited significantly higher baseline levels of inflammatory markers, including white blood cell count and CRP, whereas platelet counts were comparable between groups (Table 3). All evaluated coagulation markers, including tissue factor, PF1+2, TAT, D-dimer, and soluble P-selectin, were significantly elevated in patients with VTE. Notably, most patients classified as “hypercoagulability” exhibited abnormalities in multiple coagulation markers, suggesting that this phenotype generally reflected broad coagulation activation rather than isolated biomarker elevation. Similarly, NET-related biomarkers (cell-free DNA, calprotectin, and myeloperoxidase) were increased in the VTE group. Levels of tumor markers (CEA, CA19-9, and CA125) were also higher among patients with VTE. The optimal cutoff values for predicting baseline VTE were determined using receiver operating characteristic curve analysis and applied to subsequent analyses (Table 3).

### 3.4. Factors Associated with OS

**Figure 3:**
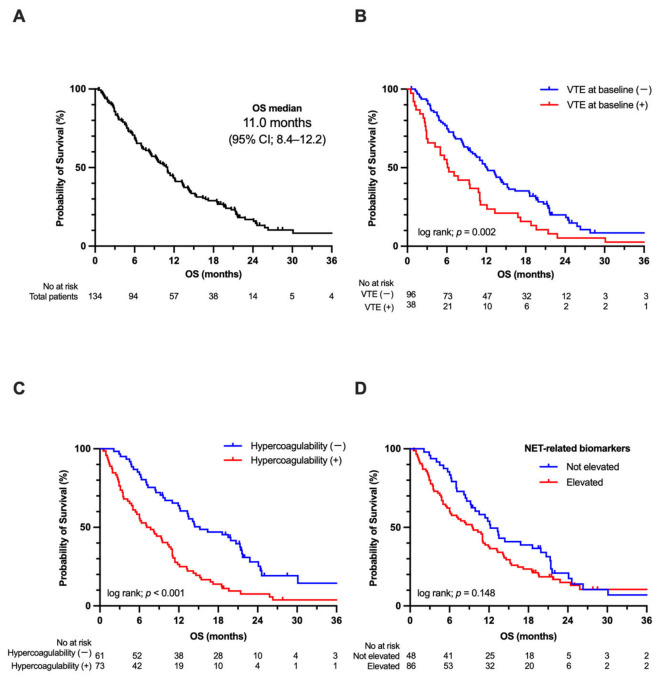
Kaplan–Meier curves for OS. (**A**) OS curve of all enrolled patients. (**B**–**D**) OS curves according to the presence of VTE (**B**), hypercoagulability (**C**) and NET-related biomarker elevation (**D**) at baseline. NETs, neutrophil extracellular traps; OS, overall survival; VTE, venous thromboembolism.

During a median follow-up period of 10.7 months (range, 0.5–40.9), 114 patients died, three were lost to follow-up, and 17 were alive at the data cutoff date. The median OS of the entire cohort was 11.0 months (95% CI, 8.5–12.2) (Figure 3A). Patients with baseline VTE had significantly shorter OS compared with those without VTE (6.2 months [95% CI, 4.2–10.8] vs. 12.1 months [95% CI, 9.6–14.6]; *p* = 0.002) (Figure 3B). Likewise, hypercoagulability was associated with markedly reduced survival (7.7 months [95% CI, 5.0–10.5] vs. 15.2 months [95% CI, 12.1–21.3]; *p* = 0.002) (Figure 3C). In contrast, patients with NET-related biomarker elevation showed a non-significant trend toward worse OS (9.5 months [95% CI, 6.0–11.4] vs. 12.2 months [95% CI, 9.3–19.9]; *p* = 0.148); this difference was not statistically significant (Figure 3D). Univariable analyses identified age ≥ 71 years, ECOG PS ≥2, pancreatic body/tail tumors, metastatic disease, systemic inflammation, anemia, and elevated CA125 as factors associated with worse OS (Table 4). Individual components of hypercoagulability, including TF, PF1+2, TAT, D-dimer, and the PIC, were each significantly associated with overall survival. Similarly, markers of systemic inflammation and NETs demonstrated prognostic relevance, whereas myeloperoxidase was not significantly associated with survival (Supplementary Table S3). Among patients with baseline VTE, survival did not differ according to symptom status, anatomical site, or thrombus localization (Supplementary Figure S1A–C). However, patients experiencing treatment delay or discontinuation because of VTE demonstrated significantly poorer survival (2.6 vs. 7.4 months; *p* = 0.002) (Supplementary Figure S1D).

Multivariable analyses were performed using two predefined models to avoid collinearity between thrombosis-related variables (Table 4). In Model 1, hypercoagulability (HR 2.03; 95% CI, 1.27–3.27) remained independently associated with poorer OS together with age (HR 1.51; 95% CI, 1.02–2.24), PS (HR 2.89; 95% CI, 1.54–5.21), tumor location (HR 2.06; 95% CI, 1.34–3.22), and hemoglobin level (HR 1.57; 95% CI, 1.05–2.35). In Model 2, baseline VTE was not independently associated with OS, whereas age (HR 1.79; 95% CI, 1.20–2.65), PS (HR 3.33; 95% CI, 1.74–6.14), tumor location (HR 1.79; 95% CI, 1.18–2.75), hemoglobin level (HR 1.58; 95% CI, 1.06–2.38), and CA125 level (HR 1.74; 95% CI, 1.05–2.85) were independent predictors of worse OS (Table 4).

### 3.5. Subgroup Analysis

**Figure 4:**
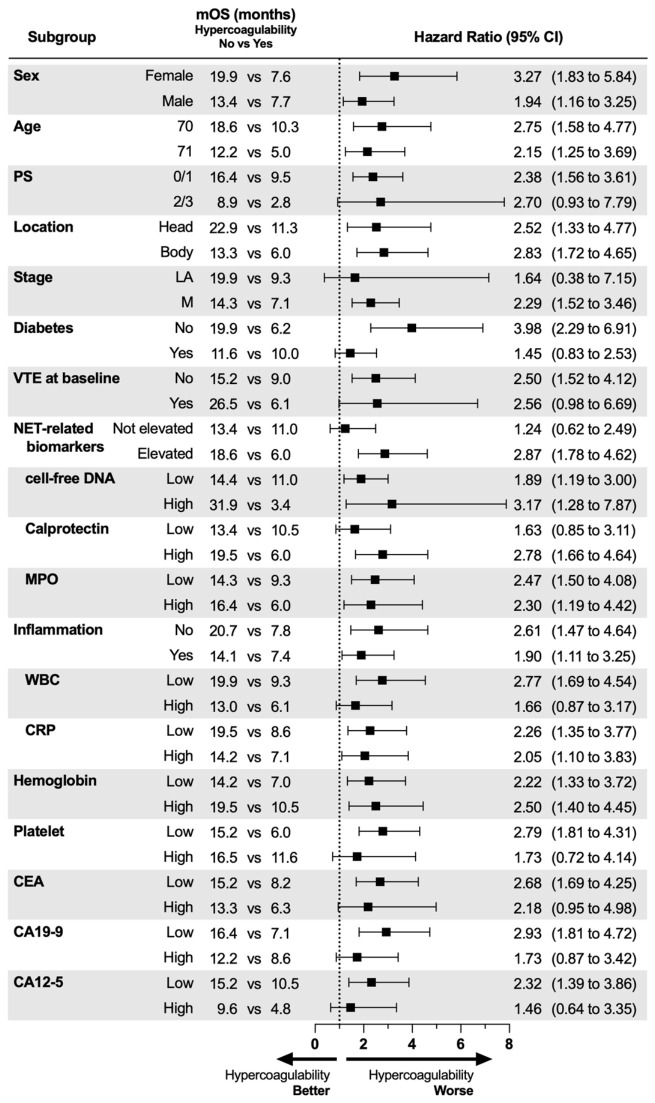
Forest plots of OS by subgroup. Forest plots of subgroup analysis by baseline characteristics and biochemical parameters for OS in all patients. Dots indicate the hazard ratio and whiskers display the 95% confidence interval. CA125, carbohydrate antigen 125; CA19-9, carbohydrate antigen 19-9; CEA, carcinoembryonic antigen; CI, confidence interval; CRP, C-reactive protein; LA, locally advanced; M, metastatic; mOS, median overall survival; MPO, myeloperoxidase; NETs, neutrophil extracellular traps; PS, performance status; VTE, venous thromboembolism; WBC, white blood cell.

The adverse prognostic impact of hypercoagulability was consistently observed across clinically relevant subgroups (Figure 4). Lack of statistical significance in selected subgroups was considered attributable to a limited sample size. These findings suggest that the prognostic impact of hypercoagulability was generally maintained across different clinical settings.

### 3.6. Association Between Anticoagulant Response and Survival

**Figure 5:**
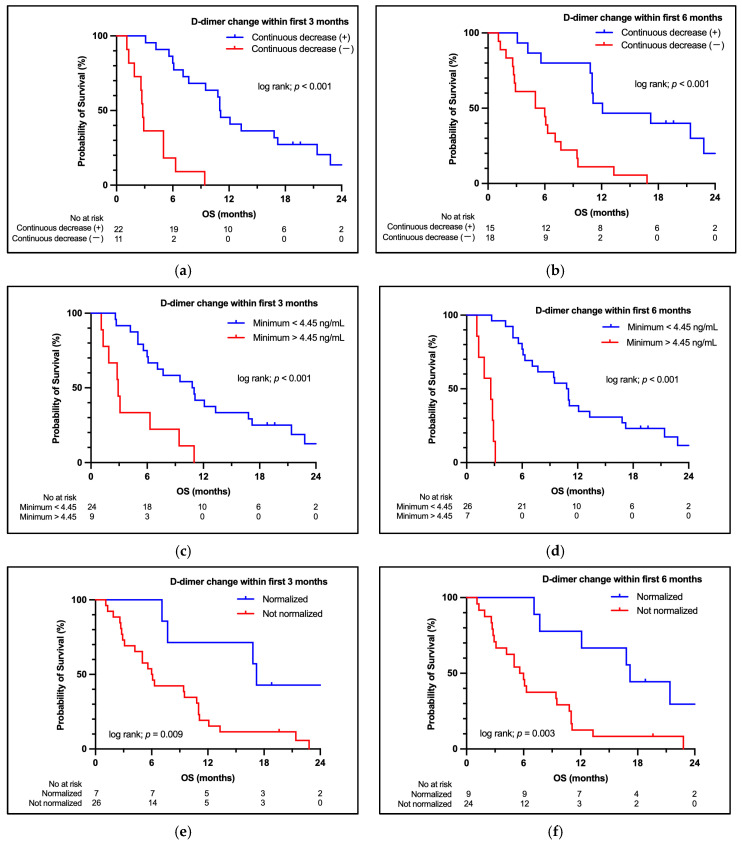
Changes in D-dimer levels and OS. Differences in OS curves associated with a continuous decrease in D-dimer level within the first 3 months (**a**) and 6 months (**b**). A continuous decrease was defined as a progressive decline in D-dimer levels at every consecutive assessment during the specified period. (**c**–**f**) OS curves associated with achievement of minimum D-dimer levels <4.45 μg/mL (**c**,**d**) and normalization (<0.5 μg/mL; (**e**,**f**)) within the first 3 months (**c**,**e**) and 6 months (**d**,**f**). OS, overall survival.

Among 33 patients with baseline VTE treated with DOACs, sustained reductions in D-dimer levels during treatment were associated with significantly prolonged OS (Figure 5a,b). Survival was significantly improved in patients whose D-dimer levels declined below the predefined cutoff value (4.45 µg/mL) or normalized within 3 or 6 months (Figure 5c–f).

## 4. Discussion

In this prospective study that incorporated systematic VTE screening, we demonstrated a substantial burden of CAT and hypercoagulability in patients with APC. NET-related biomarkers were associated with VTE development, supporting a potential link with CAT. While baseline VTE was not independently associated with survival after adjustment, hypercoagulability independently predicted OS across analyses and subgroups, suggesting that outcomes may be more closely associated with the underlying prothrombotic state than with overt thrombotic events. The consistency of these findings across clinically relevant subgroups further supports the potential applicability of hypercoagulability as a prognostic biomarker in routine clinical practice, although larger validation studies are warranted.

The prognostic relevance of CAT in APC has remained controversial, with previous studies reporting inconsistent associations with survival outcomes [10,11,13,14,15,18,19]. Most prior investigations relied on retrospectively detected or clinically apparent thrombotic events, which likely underestimated the true thrombotic burden. By prospectively applying systematic screening, our study provides a more comprehensive evaluation and indicates that hypercoagulability represents a broader disease phenotype extending beyond clinically detectable VTE. Notably, the majority of VTE events identified in our cohort were asymptomatic, likely reflecting the systematic screening strategy employed in this study. This finding suggests that a substantial proportion of thrombotic events may remain unrecognized in routine clinical practice. Early detection of occult VTE may facilitate timely initiation of anticoagulant therapy and potentially reduce thrombotic progression or complications that could interfere with the delivery of anticancer treatment. Clinically apparent VTE likely represents only the terminal manifestation of a systemic prothrombotic state driven by tumor burden, inflammation, and host–tumor interactions, whereas hypercoagulability may reflect ongoing activation of coagulation pathways associated with tumor progression [3,34]. This conceptual distinction may partly explain discrepancies among earlier reports and highlights the importance of evaluating coagulation activation as a continuous biological process rather than a binary clinical event.

Our findings highlight an emerging thromboinflammatory framework linking coagulation, inflammation, and tumor progression in APC. NET-related biomarkers were associated with coagulation activation, VTE development, and worse OS, consistent with previous reports suggesting a potential role of NETs in thrombus formation, tumor progression, metastatic dissemination, and cancer-associated inflammation [22,25,26,27]. Similarly, CA125, a tumor-derived mucin, was associated with hypercoagulability and poorer OS in our cohort, reflecting its role in platelet activation and selectin-mediated interactions [34,35]. Together, these findings suggest that NET-related pathways and tumor-derived mucins may contribute to the systemic prothrombotic state associated with aggressive pancreatic cancer biology. Collectively, our findings indicate that systemic hypercoagulability reflects underlying tumor aggressiveness in APC and may explain why markers of coagulation activation provide significant prognostic information beyond clinically detectable thrombotic events.

Importantly, among patients with baseline VTE receiving anticoagulant therapy, a decline in D-dimer levels during treatment was associated with significantly prolonged survival. This observation suggests that longitudinal changes in coagulation activity may have prognostic value beyond the occurrence of thrombotic events alone. However, because all patients in this analysis received anticoagulant therapy for established VTE, the observed association should not be interpreted as evidence that anticoagulation itself improved survival. Reduced D-dimer levels may reflect lower thrombotic burden, more favorable tumor biology, a better response to anticancer therapy, or other prognostically favorable factors. Furthermore, the observational nature of this analysis precludes exclusion of reverse causation and immortal time bias. Consistent with this interpretation, prior randomized trials in pancreatic cancer failed to demonstrate a survival benefit from the addition of anticoagulation to chemotherapy [18,36], and current guidelines do not support anticoagulation for antitumor purposes [37]. Nevertheless, serial assessment of D-dimer levels may provide clinically useful prognostic information for patients with APC and hypercoagulability, although these findings require validation in prospective studies.

Several limitations of this study should be acknowledged. First, although the present study was prospectively conducted with systematic VTE screening in all enrolled patients, it was conducted at a single institution, which may limit the generalizability of the findings. Second, anticoagulant therapy was administered according to physician discretion rather than in accordance with a predefined protocol, precluding definitive conclusions regarding the causal impact of anticoagulation on survival. Third, NET-related biomarkers were evaluated using surrogate markers rather than direct functional assays of NET formation. As such, the present findings should be interpreted as associations involving NET-related biomarkers rather than direct evidence of NET formation or NETosis-mediated mechanisms. Fourth, the cutoff values for coagulation parameters were derived from receiver operating characteristic analyses within the study cohort and therefore require external validation. Finally, although this study was prospectively conducted, the modest sample size may have increased the risk of model overfitting in multivariable analyses. In addition, the sample size has limited statistical power for the analysis of certain subgroups. Therefore, the present findings should be considered exploratory and require validation in larger multicenter cohorts. The findings are, however, being further evaluated in a multicenter study (UMIN000048619). Nevertheless, the prospective design incorporating systematic VTE screening together with comprehensive assessment of coagulation- and NET-related biomarkers represents a major strength of this study and has enabled a more accurate characterization of thromboinflammatory status in APC.

## 5. Conclusions

In conclusion, hypercoagulability emerged as a key prognostic factor in APC, whereas overt VTE alone did not independently influence survival outcomes. Our findings highlight the potential importance of thromboinflammatory activation in shaping both thrombotic risk and disease prognosis. Future multicenter studies are warranted to validate these findings and to further clarify the clinical and biological significance of hypercoagulability and NET-related pathways in APC.

## Figures and Tables

**Table 1:** Patient characteristics and prevalence of VTE.

Factors	Total		VTE (+) at Baseline			Cumulative Incidence of VTE		
	No.	(%)	No.	(%)	*p* Value	3 m	6 m	*p* Value
Total	134	(100%)	38	(28.4)		36.2%	39.7%	
Gender								
Female	67	(50.0)	22	(32.8)	0.338	38.9%	45.4%	0.106
Male	67	(50.0)	16	(23.9)		33.4%	33.4%	
Age								
≤70	74	(55.2)	18	(24.3)	0.335	32.6%	38.4%	0.769
≥71	60	(44.8)	20	(33.3)		40.5%	40.5%	
Performance status								
≤1	116	(86.6)	27	(23.3)	0.003	32.2%	36.1%	<0.001
≥2	18	(13.4)	11	(61.1)		61.1%	61.1%	
Location								
Head	51	(38.1)	10	(19.6)	0.114	31.4%	35.5%	0.142
Body/tail	83	(61.9)	28	(33.7)		39.0%	42.1%	
Disease extent								
Locally advanced	24	(17.9)	2	(8.3)	0.022	8.3%	12.9%	0.017
Metastatic	110	(82.1)	36	(32.7)		42.2%	45.6%	
Major vessel compression ^a^								
No	71	(53.0)	22	(31.0)	0.566	41.6%	43.3%	0.432
Yes	63	(47.0)	16	(25.4)		30.2%	35.6%	
Diabetes								
No	78	(58.2)	22	(28.2)	>0.999	37.1%	38.5%	0.862
Yes	56	(41.8)	16	(28.6)		34.8%	41.8%	
Prior anticoagulant use								
No	116	(86.6)	32	(27.6)	0.587	36.6%	42.9%	0.940
Yes	18	(13.4)	6	(33.3)		33.3%	40.5%	
Personal history of VTE								
No	122	(76.1)	36	(30.4)	0.508	38.2%	42.0%	0.430
Yes	12	(9.0)	2	(16.7)		25.9%	25.9%	
Blood type								
A/B/AB	102	(76.1)	31	(29.5)	0.508	37.5%	41.0%	0.223
O	32	(23.9)	7	(21.9)		31.9%	35.5%	
White blood cell count (/µL)								
≤11,000	123	(91.8)	32	(26.0)	0.075	34.4%	38.2%	0.088
>11,000	11	(8.2)	6	(54.5)		54.5%	54.5%	
Hemoglobin level (g/dL)								
<10	2	(1.5)	0	(0)	>0.999	NA		
≥10	132	(98.5)	38	(28.8)		NA		
Platelet count (×10^4^/uL)								
≤35	124	(92.5)	36	(29.0)	0.724	36.7%	39.5%	0.706
>35	10	(7.5)	2	(20.0)		30.0%	40.0%	
Body mass index (kg/m^2^)								
<35	133	(99.3)	37	(27.8)	0.284	NA		
≥35	1	(0.7)	1	(100)		NA		
D-dimer (µg/L)								
<1.44	55	(41.0)	2	(3.6)	<0.001	11.6%	13.6%	<0.001
≥1.44	78	(58.2)	36	(46.2)		51.9%	56.8%	
Soluble P-selectin (mg/L)								
<53.1	105	(78.4)	20	(19.1)	<0.001	27.0%	31.4%	<0.001
≥53.1	29	(21.6)	18	(62.1)		70.1%	70.1%	

VTE, venous thromboembolism. ^a^ Vascular invasion/compression of the portal vein and/or the superior mesenteric vein.

**Table 2:** Utility of VTE risk assessment models in APC.

Risk Assessment Models	Total		VTE (+) at Baseline			Cumulative Incidence of VTE		
	No.	(%)	No.	(%)	*p* Value	3 m	6 m	*p* Value
Khorana								
Low (≤2)	112	(83.6)	30	(26.8)	0.438	35.1%	38.2%	0.549
High (≥3)	22	(16.4)	8	(36.4)		41.3%	47.8%	
Vienna								
Low (≤2)	80	(59.7)	15	(18.8)	0.003	22.6%	24.0%	<0.001
High (≥3)	54	(40.3)	23	(42.6)		56.7%	63.6%	
PROTECHT								
Low (≤2)	112	(83.6)	30	(26.8)	0.438	35.1%	38.2%	0.549
High (≥3)	22	(16.4)	8	(36.4)		41.3%	47.8%	
CONKO								
Low (≤2)	100	(74.6)	23	(23.0)	0.027	32.2%	35.6%	0.048
High (≥3)	34	(25.4)	15	(44.1)		47.4%	52.2%	
ONKOTEV								
Low (≤2)	84	(62.7)	24	(28.6)	>0.999	36.1%	38.8%	0.404
High (≥3)	50	(37.3)	14	(28.0)		36.2%	41.2%	
COMPASS								
Low (≤6)	64	(47.8)	15	(23.4)	0.254	31.6%	34.8%	0.278
High (≥7)	70	(52.2)	23	(32.9)		40.7%	44.7%	
COMPASS								
Low (≤10)	85	(63.4)	20	(23.5)	0.115	30.7%	34.7%	0.076
High (≥11)	49	(36.6)	18	(36.7)		45.6%	48.4%	
RIETE								
Low (≤3)	86	(64.2)	17	(19.8)	<0.001	29.2%	33.1%	0.007
Intermediate (4–6)	30	(22.4)	10	(33.3)		40.6%	44.9%	
High (≥7)	18	(13.4)	11	(61.1)		61.1%	61.1%	

VTE, venous thromboembolism.

**Table 3:** Biochemical/coagulation profile of VTE.

Parameter	VTE (+) Mean ± SEM			VTE (−) Mean ± SEM			*p* Value	Cut-Off	AUC	(95% CI)
White blood cell count (/µL)	8472	±	560	7006	±	227	0.004	7955	0.614	(0.499–0.730)
Hemoglobin level (g/dL)	12.7	±	0.2	13.3	±	0.2	0.047	13.1	0.630	(0.526–0.735)
Platelet count (×10^4^/uL)	21.9	±	1.1	24.4	±	0.9	0.106	28.2	0.566	(0.461–0.672)
CRP (mg/dL)	3.73	±	0.96	1.49	±	0.33	0.006	0.90	0.664	(0.560–0.768)
Tissue factor (pg/mL)	131.2	±	38.5	31.7	±	4.8	<0.001	41.0	0.728	(0.630–0.827)
PF1+2 (pmol/mL)	875.0	±	102.5	326.7	±	21.8	<0.001	509.0	0.868	(0.805–0.930)
TAT (ng/mL)	12.07	±	1.72	5.34	±	1.12	0.002	8.1	0.788	(0.703–0.874)
D-dimer (ng/mL)	17.89	±	2.54	2.63	±	0.38	<0.001	4.45	0.894	(0.829–0.959)
Soluble P-selectin (ng/mL)	56.59	±	5.05	31.26	±	2.01	<0.001	66.9	0.740	(0.643–0.837)
PIC (µg/mL)	4.51	±	0.68	1.45	±	0.13	<0.001	1.85	0.788	(0.693–0.884)
Cell-free DNA (ng/mL)	1.62	±	0.43	0.31	±	0.04	<0.001	0.58	0.867	(0.794–0.940)
Calprotectin (ng/mL)	838.1	±	26.6	718.6	±	15.4	<0.001	721.0	0.718	(0.619–0.816)
Myeloperoxidase (ng/mL)	298.3	±	36.6	182.4	±	14.9	<0.001	185.5	0.669	(0.561–0.776)
CEA (ng/mL)	77.2	±	37.2	10.4	±	1.9	0.005	14.6	0.685	(0.580–0.791)
CA19-9 (IU/mL)	438,700	±	182,973	12,996	±	5614	<0.001	8606	0.675	(0.563–0.788)
CA125 (IU/mL)	132.6	±	18.7	50.1	±	8.4	<0.001	74.5	0.759	(0.668–0.849)

AUC, area under the curve; CEA, carcinoembryonic antigen; CA125, carbohydrate antigen 125; CA19-9, carbohydrate antigen 19-9; CI, confidence interval; CRP, C-reactive protein; DNA, deoxyribonucleic acid; PF1+2, prothrombin fragment 1+2; PIC, plasmin–α2 plasmin inhibitor complex; SEM, standard error of the mean; TAT, thrombin–antithrombin III complex.

**Table 4:** Univariate and multivariate analyses of OS.

Factor	No.	Univariate			Multivariate Model 1		Multivariate Model 2	
		mOS	(95% CI)	*p* Value	HR	(95% CI)	HR	(95% CI)
Total	134	11.0 m	(8.5 to 12.2)					
Gender								
Female	67	11.8 m	(8.4 to 14.3)	0.170				
Male	67	10.5 m	(7.0 to 12.1)					
Age								
≤70	74	12.1 m	(10.8 to 15.2)	0.006	Ref		Ref	
≥71	60	7.0 m	(5.0 to 11.0)		1.51	(1.02 to 2.24)	1.79	(1.20 to 2.65)
PS								
≤1	116	11.8 m	(9.9 to 14.1)	<0.001	Ref		Ref	
≥2	18	2.9 m	(2.4 to 5.0)		2.89	(1.54 to 5.21)	3.33	(1.74 to 6.14)
Location								
Head	51	14.6 m	(11.4 to 20.7)	<0.001	Ref		Ref	
Body/tail	83	8.4 m	(6.0 to 10.5)		2.06	(1.34 to 3.22)	1.79	(1.18 to 2.75)
Disease extent								
LA	24	15.2 m	(9.3 to 24.1)	0.048	Ref		Ref	
Metastatic	110	9.9 m	(7.3 to 11.4)		1.36	(0.77 to 2.51)	1.83	(1.06 to 3.28)
Diabetes								
No	78	11.4 m	(7.0 to 14.6)	0.140				
Yes	56	10.7 m	(8.2 to 12.2)					
VTE at baseline								
No	96	12.1 m	(9.6 to 14.6)	0.002	-		Ref	
Yes	38	6.2 m	(4.2 to 10.8)		-		0.84	(0.52 to 1.33)
Hypercoagulability								
No	61	15.2 m	(12.1 to 21.3)	<0.001	Ref		-	
Yes	73	7.7 m	(5.0 to 10.5)		2.03	(1.27 to 3.27)	-	
NET-related biomarkers								
Not elevated	48	12.2 m	(9.3 to 19.9)	0.148				
Elevated	86	9.5 m	(6.0 to 11.4)					
Inflammation								
No	71	11.1 m	(8.6 to 18.6)	0.024	Ref		Ref	
Yes	63	9.4 m	(6.3 to 11.9)		0.85	(0.55 to 1.30)	0.92	(0.60 to 1.41)
Hemoglobin (g/dL)								
Low	69	8.3 m	(6.1 to 11.1)	0.030	1.57	(1.05 to 2.35)	1.58	(1.06 to 2.38)
High	65	13.2 m	(10.5 to 15.4)		Ref		Ref	
Platelet (×10^4^/L)								
Low	108	9.5 m	(7.1 to 11.4)	0.288				
High	26	13.9 m	(11.4 to 18.6)					
CEA (ng/mL)								
Low	102	11.0 m	(8.6 to 13.5)	0.143				
High	32	8.7 m	(5.6 to 12.1)					
CA19-9 (IU/mL)								
Low	94	11.0 m	(8.3 to 14.2)	0.107				
High	40	9.6 m	(5.0 to 12.1)					
CA125 (IU/mL)								
Low	92	13.2 m	(10.5 to 15.2)	<0.001	Ref		Ref	
High	42	4.9 m	(2.9 to 7.0)		1.28	(0.77 to 2.12)	1.74	(1.05 to 2.85)

CA125, carbohydrate antigen 125; CA19-9, carbohydrate antigen 19-9; CEA, carcinoembryonic antigen; CI, confidence interval; HR, hazard ratio; LA, locally advanced; NETs, neutrophil extracellular traps; PS, performance status; VTE, venous thromboembolism.

## Data Availability

The raw data supporting the conclusions of this article will be made available by the authors upon request.

## Supplementary Materials

The following supporting information can be downloaded at https://www.mdpi.com/article/10.3390/cancers18132120/s1, Figure S1: Type of VTE and OS. Table S1: Scoring system for risk assessment models of cancer-associated thrombosis. Table S2: Characteristics and treatment of VTE. Table S3: Biochemical parameters and OS.

## Abbreviations

The following abbreviations are used in this manuscript:

APC	Advanced pancreatic cancer
CAT	Cancer-associated thrombosis
CA125	Carbohydrate antigen 125
CA19-9	Carbohydrate antigen 19-9
CEA	Carcinoembryonic antigen
CIs	Confidence intervals
CRP	C-reactive protein
CT	Computed tomography
DOACs	Direct oral anticoagulants
ECOG	Eastern Cooperative Oncology Group
ELIZAs	Enzyme-linked immunosorbent assays
HRs	Hazard ratios
NETs	Neutrophil extracellular traps
OS	Overall survival
PF1+2	Prothrombin fragment 1+2
PIC	Plasmin–α2 plasmin inhibitor complex
PS	Performance status
RAMs	Risk assessment models
TAT	Thrombin–antithrombin III complex
VTE	Venous thromboembolism
