# Supplementary material for: Hypercoagulability Predicts Survival and Reflects NET-Associated Thromboinflammation in Advanced Pancreatic Cancer
Source: Cancers (Basel). 2026 Jun 30;18(13):2120. doi: 10.3390/cancers18132120 (PMC13359987; doi:10.3390/cancers18132120)
Supplement: Supplementary file 1 [file cancers-18-02120-s001.zip › cancers-4330896 supplementary tables.pdf]

**Supplementary Table S1.** Scoring system for risk assessment models of cancer-associated thrombosis

| Variable | Khorana Ref [5] | Vienna Ref [28] | PROTECHT Ref [29] | CONKO Ref [30] | ONKOTEV Ref [31] | COMPASS Ref [32] | RIETE Ref [33] |
|---|---|---|---|---|---|---|---|
| Cancer site – very high risk (pancreas, stomach) | 2 | 2 | 2 | 2 | – | – | – |
| Cancer site – high risk (lung, lymphoma, gynecologic, bladder, testicular) | 1 | 1 | 1 | 1 | – | – | – |
| Platelet count > 35 $\times 10^4/\mu L$ | 1 | 1 | 1 | 1 | – | 2 | – |
| Platelet count ≤ 16.5 $\times 10^4/\mu L$ | – | – | – | – | – | – | 2 |
| Hemoglobin < 10 g/dL or use of red cell growth factor | 1 | 1 | 1 | 1 | – | – | – |
| White blood cell count > 11000 /μL | 1 | 1 | 1 | 1 | – | – | – |
| White blood cell count > 11500 /μL | – | – | – | – | – | – | 4 |
| Body mass index ≥ 35 kg/m² | 1 | 1 | 1 | – | – | – | – |
| Body mass index < 18.5 kg/m² | – | – | – | – | – | – | 3 |
| D-dimer ≥ 1.44 μg/L | – | 1 | – | – | – | – | – |
| Soluble P-selectin ≥ 53.1 mg/L | – | 1 | – | – | – | – | – |
| Platinum chemotherapy | – | – | 1 | – | – | – | – |
| Gemcitabine chemotherapy | – | – | 1 | – | – | – | – |
| Performance status ≥ 2 | – | – | – | 1 | – | – | – |
| Khorana score ≥ 2 | – | – | – | – | 1 | – | – |
| Metastatic disease/Advanced stage | – | – | – | – | 1 | 2 | 3 |
| History of VTE/PE | – | – | – | – | 1 | 1 | 2 |
| Tumor vascular/lymphatic compression | – | – | – | – | 1 | – | – |
| Central venous catheter | – | – | – | – | – | 3 | – |
| Anthracycline therapy | – | – | – | – | – | 6 | – |
| Cancer diagnosis ≤ 6 months | – | – | – | – | – | 4 | – |
| Cardiovascular risk factors | – | – | – | – | – | 5 | – |
| Recent hospitalization / immobility | – | – | – | – | – | 5 | 3 |

PE, pulmonary embolism; VTE, venous thromboembolism.

**Supplementary Table S2.** Characteristics and treatment of VTE

| Parameter | Baseline No. (%) | Follow-up No. (%) |
|---|---|---|
| All VTE events | 38 | 22 |
| Type of VTE | | |
| Symptomatic | 6 (15.8) | 5 (22.7) |
| Asymptomatic | 32 (84.2) | 17 (77.3) |
| Site of VTE | | |
| DVT | 38 (100.0) | 22 (100.0) |
| Proximal | 7 (18.4) | 3 (13.6) |
| Distal | 31 (81.6) | 19 (86.4) |
| PE ± DVT | 4 (10.5) | 1 (4.5) |
| Adverse event with VTE | | |
| Unplanned hospitalization | 7 (18.4) | 4 (18.2) |
| Treatment delay/discontinuation[a] | 4 (10.5) | 1 (4.5) |
| VTE-related death | 0 (0.0) | 0 (0.0) |
| Anticoagulation therapy | | |
| Unfractionated heparin (induction) | 6 (15.8) | 4 (18.2) |
| DOAC | 33 (86.8) | 21 (95.5%) |
| Antiplatelet agent | 2 (5.3) | 0 (0.0) |
| VKA | 1 (2.6) | 0 (0.0) |
| None | 2 (5.3) | 1 (4.5) |

DOAC, direct oral anticoagulant; DVT, deep venous thrombosis; PE, pulmonary embolism; VTE, venous thromboembolism; VKA, vitamin K antagonist.

[a] Delay for > 4 weeks or discontinuation of anti-cancer treatment due to VTE.

**Supplementary Table S3.** Biochemical parameters and OS

| Parameter | No. | mOS | (95% CI) | *P* value |
|---|---|---|---|---|
| **Hypercoagulability** | | | | |
| Tissue factor (pg/mL) | | | | |
| Low | 92 | 12.1 m | (10.3 to 15.2) | < 0.001 |
| High | 42 | 6.1 m | (4.2 to 9.4) | |
| PF1+2 (pmol/mL) | | | | |
| Low | 92 | 13.4 m | (10.3 to 15.4) | < 0.001 |
| High | 42 | 5.0 m | (2.9 to 7.1) | |
| TAT (ng/mL) | | | | |
| Low | 108 | 12.1 m | (9.9 to 14.4) | < 0.001 |
| High | 26 | 6.2 m | (2.9 to 9.4) | |
| D-dimer (ng/mL) | | | | |
| Low | 89 | 14.1 m | (11.4 to 18.5) | < 0.001 |
| High | 45 | 5.6 m | (3.1 to 7.1) | |
| soluble P-selectin (ng/mL) | | | | |
| Low | 113 | 11.9 m | (10.3 to 14.2) | < 0.001 |
| High | 21 | 5.4 m | (1.9 to 7.1) | |
| PIC (μg/mL) | | | | |
| Low | 90 | 13.3 m | (10.5 to 17.2) | < 0.001 |
| High | 44 | 6.0 m | (4.2 to 9.4) | |
| **NETs-related biomarkers** | | | | |
| cell-free DNA (ng/mL) | | | | |
| Low | 99 | 12.2 m | (10.5 to 14.6) | < 0.001 |
| High | 35 | 4.8 m | (2.8 to 6.3) | |
| Calprotectin (ng/mL) | | | | |
| Low | 60 | 12.2 m | (9.2 to 18.6) | 0.038 |
| High | 74 | 9.5 m | (5.6 to 11.4) | |
| Myeloperoxidase (ng/mL) | | | | |
| Low | 87 | 11.8 m | (9.3 to 14.3) | 0.151 |
| High | 47 | 8.3 m | (5.0 to 11.4) | |
| **Inflammation** | | | | |
| White blood cell count (/μL) | | | | |
| Low | 91 | 11.4 m | (9.5 to 14.3) | 0.006 |
| High | 43 | 7.3 m | (5.4 to 11.9) | |
| CRP (mg/dL) | | | | |
| Low | 85 | 11.4 m | (8.6 to 15.2) | 0.012 |
| High | 49 | 9.3 m | (5.0 to 11.4) | |

CI, confidence interval; CRP, C-reactive protein; DNA, deoxyribonucleic acid; mOS, median overall survival; NETs, neutrophil extracellular traps; PF1+2, prothrombin fragment 1+2; PIC, plasmin-$\alpha$2 plasmin inhibitor complex; TAT, thrombin-antithrombin III complex.
