# Supplementary figures and images for: Hypercoagulability Predicts Survival and Reflects NET-Associated Thromboinflammation in Advanced Pancreatic Cancer
Source: Cancers (Basel). 2026 Jun 30;18(13):2120. doi: 10.3390/cancers18132120 (PMC13359987; doi:10.3390/cancers18132120)

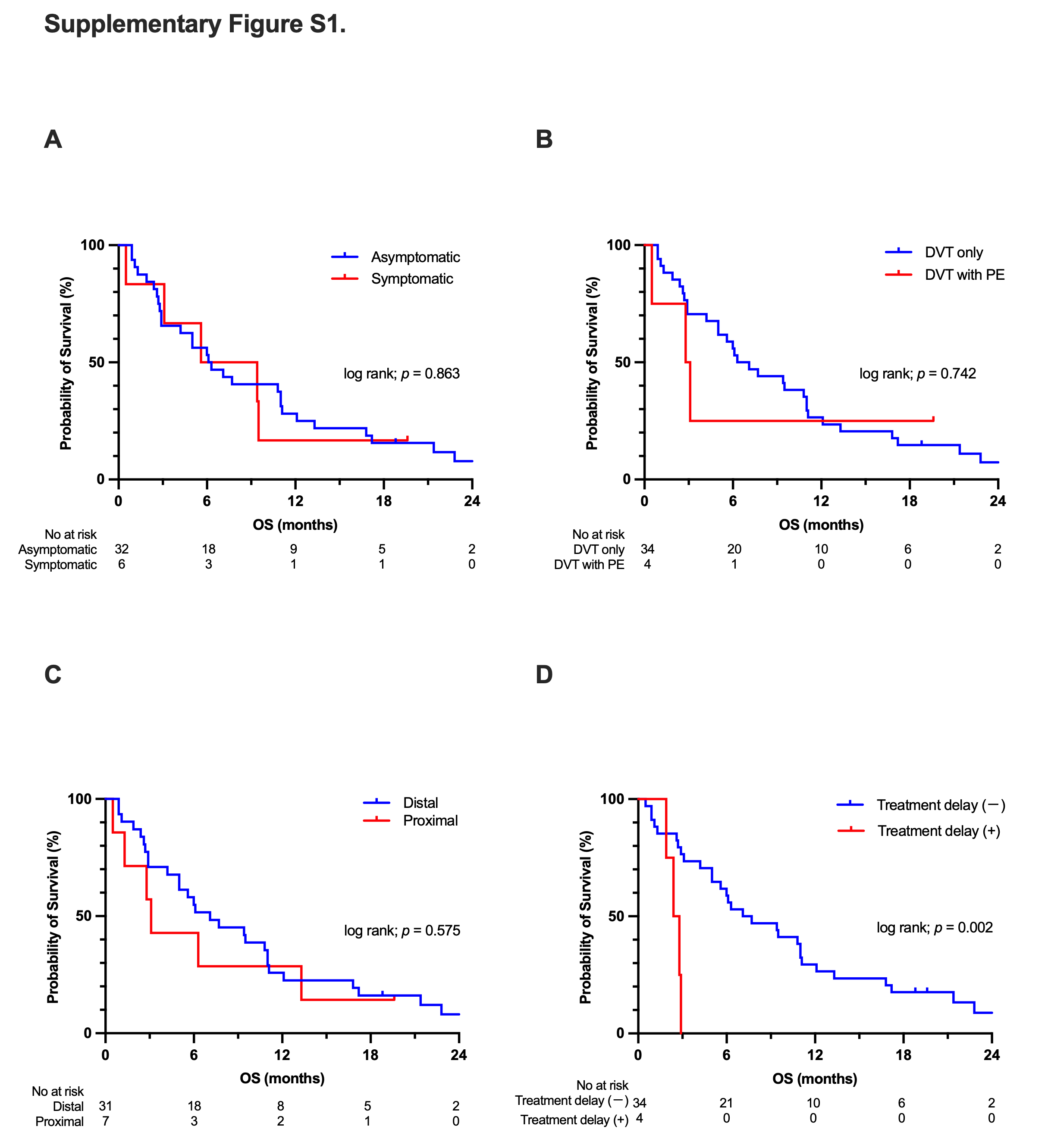

Supplement: Supplementary file 1 [file cancers-18-02120-s001.zip › cancers-4330896 FigS1.tiff]
